# Prevalence and genotyping of *Pseudomonas aeruginosa* from food and human sources

**DOI:** 10.1038/s41598-026-37559-y

**Published:** 2026-02-17

**Authors:** Walid S. Mousa, Eman E. Abdeen, Hanem F. El-Gendy, Reem M. Alsaadawy, Mary M. Hana

**Affiliations:** 1 Department of Medicine and Infectious Diseases, Faculty of Veterinary Medicine, University of Sadat City, Sadat City, 32958 Egypt; 2 Department of Bacteriology, Mycology and Immunology, Faculty of Veterinary Medicine, University of Sadat City, Sadat City, 32958 Egypt; 3 Department of Pharmacology, Faculty of Veterinary Medicine, University of Sadat City, Sadat City, 32897 Egypt; 4 Department of Zoonoses, Faculty of Veterinary Medicine, Assiut University, Assiut, 71526 Egypt; 5 Technical Veterinary at the laboratory of the Haripur hospital, Sadat City, 32958 Egypt

**Keywords:** Antibiotic resistance, ERIC-PCR, Food, Human samples, *P. aeruginosa*, Virulence, Diseases, Microbiology, Molecular biology

## Abstract

*Pseudomonas aeruginosa* is a common pathogen of a wide range of nosocomial infections in humans, as well as foodborne illnesses. The current study focuses on the molecular genotyping of *P. aeruginosa* recovered from human, water, and food specimens. A total of 350 samples, fifty samples each from human, tap water, fish swamp, chicken meat, minced meat, raw milk, and hospital surface from the Menoufiya governorate, Egypt. *P. aeruginosa* was detected in 14.28%, including human (26%), tap water (18%), fish swamp (18%), chicken meat (12%), minced meat (10%), raw milk (16%), and hospital surface (0%). The results of testing of 50 *P. aeruginosa* isolates against sixteen antibiotics revealed a relatively high antibiotic resistance for Amoxicillin (100%), Erythromycin (98%), Cephradine (90%), Colistin (82%), Oxytetracyclin, (79%), Chloramphenicol (70%), Doxycycline (70%), and Kanamycin (62%) and high susceptibility for Imipenem (96%), Apramycin (94%), Amikacin (90%), Norfloxacin (78%), Sulphamethoxazol (86%), Enrofloxacin (64%), and Ofloxacin (60%). Furthermore, PCR was successfully amplified for the *tox*A, *exo*S, and *opr*L virulence genes at 396, 118, and 504 bp, respectively, as well as amplifying the *erm*B, *pel*A, *bla*TEM, and *tet*A resistance genes at 639, 786, 516, and 570 bp, respectively. The dendrogram investigation by ERIC-PCR of 10 clinical *P. aeruginosa* isolates revealed two main clusters and 10 different ERIC-PCR patterns. The presence of *P. aeruginosa* isolates in food may represent a potential public health concern, with the need for further epidemiological studies, as well as whole-genome sequencing and correlations of *P. aeruginosa* in water, food samples, and human infections.

**Supplementary Information:**

The online version contains supplementary material available at 10.1038/s41598-026-37559-y.

## Introduction

*Pseudomonas aeruginosa* is an abundant environmental pathogen found in soil and water. It is a main reason for both cystic fibrosis and nosocomial infections in humans^1^. Definitely, drinking water is consistently regarded as an important environmental habitat for *P. aeruginosa*^2^. In addition, severe illnesses resulted from the consumption of contaminated milk products^3^. Many *Pseudomonas* species, mostly *P. aeruginosa*,* have* been incriminated as the main relevant agents of numerous illnesses in both veterinary and medical concerns^4^.

Globally, serious nosocomial infections and malignant syndromes are caused by invasive and opportunistic *P. aeruginosa*, with significant increases in deaths and morbidity rates in humans^5^. Hence, *P. aeruginosa* is an obligate aerobe pathogen that can synthesize arginine, proliferate in anaerobic conditions, and this makes it the most plentiful organism on earth^6^. This organism has been associated with various disease syndromes, including urinary tract infections, respiratory tract infections, pneumonia, and wound infections^7^. In addition, McCabe-Sellers and Beattie^8^ reported *P. aeruginosa* as a causal agent of numerous food-borne illnesses. Additionally, *P. aeruginosa* is primarily a food contaminant and spoilage organism, with occasional involvement in food-associated infections as a result of the consumption of contaminated food and poor hygiene procedures^9^.

Interestingly, the emergence of resistant *P. aeruginosa* isolates to various antibiotics has led to widespread infections in numerous organs such as the respiratory, urinary, and nervous tissues, resulting in substantial morbidity and deaths, and emerging as a nosocomial pathogen, particularly in immunocompromised patients, posing a life-threatening complaint^10,11^. Furthermore, it was widely recognized that *P. aeruginosa’s* pathogenicity is largely triggered by various virulence elements^5^. *P. aeruginosa* pathogenicity is attributed to the secretion of various invasive, toxigenic extracellular components, which exert substantial effects on the disease severity^12^. Moreover, the virulence activity is related to many determinants comprising enzymes such as monooxygenase, proteases, alginate, phospholipases, pyocyanin pigment, as well as three secretory systems and toxins, which interfere with the host cellular alleyways^13^. Additionally, other virulence elements such as exotoxins, proteases, elastases, alginate, phenazine that are encoded by *tox*A, *tox*R, *lec*A, *plc*H, *las*B, and *phz*A1 genes, respectively, and diverse pigments, thus contribute a role in the disease severity^12,14^.

Furthermore, *P. aeruginosa* can create biofilms in a variety of sites, reducing antibiotic treatment effectiveness, which in turn motivates the development of chronic illnesses^15^. This pathogen is considered the extremely adaptable microorganisms that have the capability to persevere in different environments through its involved genomic elements.

Another critical aspect of *P. aeruginosa* complications is the emergence of antimicrobial resistance (AMR), which frequently produces obstacles in controlling infections, as well as developing rapid resistance as a result of inherent and developed resistance elements due to abuse or misuse of antimicrobials^16^. Multidrug-resistant *P. aeruginosa* multiply by establishing biofilms, which can result in harmful nosocomial infections in hospitalized patients^13^. Antibiotics become less effective against bacteria that produce biofilms, which is a crucial outcome in the combat against *P. aeruginosa* strains that produce biofilms^17^.

Genetic characterization through molecular typing methods offers quick and reliable tools that are useful for tracking the origins and subsequently preventing *P. aeruginosa* infections across the drinking water chain^1^. Interestingly, molecular typing is a tool for genetic assortment that determines the crucial phenotypic characters such as virulence determinants, pathogenicity, specificity, and AMR^18^. Enterobacterial Repetitive Intergenic Consensus Polymerase Chain Reaction (ERIC-PCR) is a Gram-negative enteric bacteria-specific repetitive extragenic palindromic PCR (rep-PCR) technique that aims for highly specific repetitive sequence components^19^. An Australian study used ERIC-PCR for genotyping of *P. aeruginosa* isolates and to study their epidemiology^20^. In a previous survey, ERIC-PCR was reported as a superior tool for classifying *P. aeruginosa* isolates than other molecular tools^21^. The use of repeated intergenic consensus sequences is the primary basis for the ERIC PCR method, providing more sensitive results for bacterial typing than phenotypic assays^22^.

The excessive existence of different food products of animal origin in Egypt without following the health inspection rules has led to contamination by several bacterial species, including *P. aeruginosa*. Consequently, this study spot highlights on the isolation rate, antimicrobial susceptibility, and application of ERIC-PCR for genetic characterization of *P. aeruginous* recovered from human and food products, as well as highlights the public health threat and the portability of resistance genes transmitting to humans via the food chain. This study uniquely highlights the potential risk of *P. aeruginosa* transmission through the food chain by integrating data from food products, hospital environmental surfaces, and human sources within the same regional setting. To the best of our knowledge, this study represents the first report of ERIC-PCR genotyping of *P. aeruginosa* isolates recovered from food and human samples in Menoufiya Governorate. In addition to this regional novelty, the study’s originality also lies in its combined sampling strategy, which integrates food products, hospital environmental surfaces, and human sources within the same investigation.

## Results

### Isolation rate of *P. aeruginosa* from food and human specimens

Examination of 350 samples from human pus (50), tap water (50), fish swab (50), chicken meat (50), minced meat (50), raw milk (50), and hospital surfaces (50) revealed that *P. aeruginosa* was isolated with a 50 (14.28%) isolation rate. All positive samples showed characteristic growth appearance on cetrimide agar medium. As described in Table 1, the prevalence of *P. aeruginosa* isolated from human, tap water, fish swamp, chicken meat, minced meat, raw milk, and hospital surface was 26, 18, 18, 12, 10, 16, and 0%, respectively.

### Biochemical and virulence features of the *P. aeruginosa* isolates

Next, we confirmed the isolated *P. aeruginosa* isolates through biochemical and enzymatic activity. The results revealed that all isolates were oxidase positive, TSI (red/red) (K/K) gas negative, urease test negative, indole negative, citrate positive, and catalase positive. Also, all *P. aeruginosa* isolates that gave characteristic colony morphology in cetrimide agar (yellow-green to blue color) were tested for their pathogenicity by culturing on Congo red medium. The results demonstrated that the biofilm phenotypes accounted for 30% (*n* = 15), being distributed as follows: 33.33% (*n* = 5) strong biofilm; 46.6% (*n* = 7) moderate biofilm; 20% (*n* = 3) weak biofilm, while 70% of isolates (*n* = 35) were recognized as non-biofilm producers. Collectively, all *P. aeruginosa* isolates of food and human origin were found to carry potential virulence activities.

### Antimicrobial profile of *P. aeruginosa* isolates from food products and human samples

The antimicrobial susceptibility of tested *P. aeruginosa* isolates from food products and human origin. The results in Table 2 revealed a high level of antibiotic resistance as follows: amoxicillin, erythromycin, cephradine, colistin, oxytetracyclin, chloramphenicol, Doxycycline, and Kanamycin with 100, 98, 90, 82, 79, 70, 70, and 62%, respectively. Meanwhile, Imipenem, apramycin, amikacin, norfloxacin, sulphamethoxazole, enrofloxacin, and ofloxacin exhibited high susceptibility with 96, 94, 90, 78, 86, 64, and 60%, respectively. Together, the tested *P. aeruginosa* isolates displayed a high degree of AMR from food products and human origin.

### Phenotypic resistance profile of *P. aeruginosa* from food and human samples

Fifty *P. aeruginosa* isolates showed multidrug resistance (MDR) against 16 different antibiotics from ten antimicrobial groups. The results of the MDR profile are presented in Table 3. Notably, all 16 antibiotics from ten antimicrobial groups were found to be resistant by only one isolate. Additionally, one isolate exhibited resistance against 15 antibiotics from nine different antimicrobial groups. Eleven *P. aeruginosa* isolates displayed resistance against 14 different antibiotics from eight different antimicrobials. Nine *P. aeruginosa* isolates demonstrated resistance to 11 antibiotic groups from seven different antimicrobials. Another 9 *P. aeruginosa* isolates displayed resistance to 8 antibiotics from six antimicrobials.

Besides, we observed four *P. aeruginosa* isolates displaying MDR to 7 antibiotics from five antimicrobial classes. Only two and five *P. aeruginosa* were found to be MDR to four and three antimicrobials, respectively. Collectively, almost all of the *P. aeruginosa* 42 (84%) isolates from human animal food origin showed MDR with an AMR index ranging from 0.25 to 1.

### Molecular characterization of virulence and antibiotic resistance genes of *P. aeruginosa* isolates

Regarding the molecular characterization of the *P. aeruginosa* strains, certain virulence and antibiotic resistance genes were tested among the selected 15 *P. aeruginosa* strains, which were biofilm producers. The PCR method successfully amplified 396, 118, and 504 nucleotide fragments of the *tox*A, *exo*S, and *opr*L virulence genes of *P. aeruginosa* strains, respectively (Supplementary Fig. 1–3). In addition to successfully amplifying 639, 786, 516, and 570 nucleotide fragments of the *ermB*, *pel*A, *bla*TEM, and *tet*A genes of *P. aeruginosa* isolates, respectively (Supplementary Fig. 4–7). The prevalence of virulence and resistance genes in the tested isolates from different sources is shown in Table 4.

### ERIC-PCR for genotyping of *P. aeruginosa* isolates

**Fig. 1:**
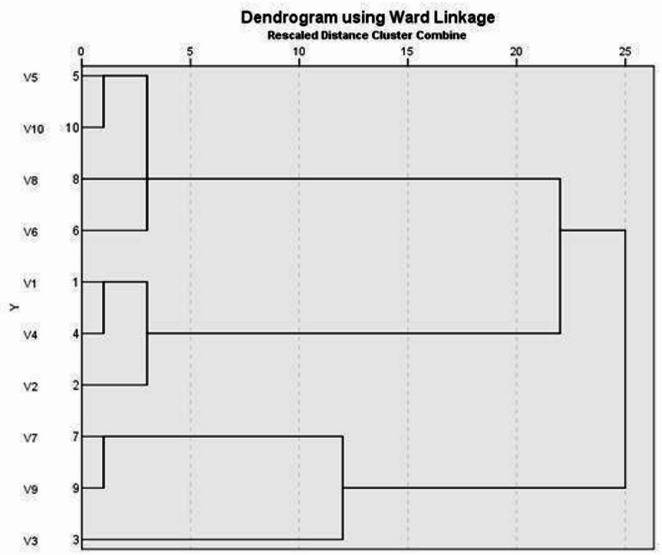
Dendrogram analysis for *Pseudomonas* aeruginosa isolates by ERIC-PCR.

Supplementary Fig. 8 shows ten clinical *P. aeruginosa* isolates amplified by ERIC-PCR bands that ranged from three to seven bands. Moreover, the molecular fragment size of the PCR products ranged from 100 to 2000 bp, with a common fragment size range, 100–1000 bp, and also 1000–2000 bp, and similarity index ranges (0.18–0.86). The results of dendrogram analysis of ten *P. aeruginosa* isolates were categorized into 2 main clusters. Moreover, 10 different ERIC patterns were generated. Cluster 1 was divided into 2 sub-clusters, the first one containing 4 serotypes from (water, milk, chicken, and fish) sources. The second sub-cluster contains 3 serotypes from different sources (human and water). Cluster II contains 3 serotypes of different sources (human, minced meat, and fish), as shown in Fig. 1.

## Discussion

*P. aeruginosa*is a frequent and common hospital-related pathogen, demonstrative in pneumonia, urinary tract, and post-surgical infections. According to Weiner et al.^23^, about 7% of health illnesses are due to *P. aeruginosa*. Our findings reported an overall isolation rate of 14.28% of *P. aeruginosa* from food and human specimens, particularly from human, tap water, fish swamp, chicken meat, minced meat, raw milk, and hospital surface, with rates of 26%,18%, 18%, 12%, 10%, 16%, and 0%, respectively. Similar international findings reported an overall *P. aeruginosa* prevalence rate of 16.2% − 23% in intensive care units, with predominance of the respiratory source^24^. Moreover, similar finding was reported from different human samples from burns, wounds, urine, and throat swabs, with prevalence rates of 22%, 9.5%, 6.9%, and 6.6%, respectively, out of 156 clinical samples.

Concerning the predominance of *P. aeruginosa* among food products, lower prevalence of *P. aeruginosa* was recorded in fresh minced meat (5%), fresh sausage (2%), and frozen beef burger (1%) samples in a previous Egyptian study^25^. In addition, Jawher and Hasan^26^ reported the predominance of *P. aeruginosa*in chicken, beef, and mutton meat, with detection rates of 22%, 14%, and 6%, respectively. Similarly, in a West African survey, Benie et al.^27^ found a high prevalence of *P. aeruginosa* in bovine meat (53.04%), fresh fish (37.69%), and smoked fish (23.57%). These findings collectively indicate widespread contamination of *P. aeruginosa* across various meat types, including camel meat (80%)^28^, chicken meat (46.7%)^29^, frozen imported meat (6.67%)^30^, and retail meat (3%)^31^. In a similar line, the current study found that *P. aeruginosa*was prevalent in raw milk at a rate of 16% while these results were higher than those obtained by Benie et al.^27^ from subclinical mastitis milk (5.4%) in Pengal.

Meanwhile, our results had a lower prevalence than those obtained by Aziz et al.^32^, who recovered 70% and 24% from milk and milk tanks, respectively. In an Egyptian study, Ibrahim et al.^33^ showed that *P. aeruginosa* was prevalent in milk and dairy products, with a prevalence rate of 22.5% as well as rates of 48%, 16%, 18%, and 8% from raw milk, yoghurt, soft cheese, and Karish cheese, respectively. In addition, our findings reported that the prevalence of *P. aeruginosa*from tap water was 18%. A reported study by Rezaloo et al.^34^ revealed a 20% recovery rate of *P. aeruginosa* from bovine frozen meat and lower in sausage (2%). The disparity in the spreading and prevalence of *P. aeruginosa* between different studies may be recognized by the ability of adaptation of *P. aeruginosa* isolates to the adverse ecological and growth conditions^35^.

Noteworthy, the testing of 50 *P. aeruginosa*strains revealed a high level of antibiotic resistance profile for Amoxicillin, Erythromycin, Cephradine, Colistin, Oxytetracyclin, Chloramphenicol, Doxycycline, and Kanamycin, while high susceptibility for Imipenem, Apramycin, Amikacin, Norfloxacin, Sulphamethoxazole, Enrofloxacin, and Ofloxacin. This was in consistent with Rezaloo et al.^34^, who detected high resistance rates against ampicillin, penicillin, tetracycline, cefoxitin, gentamicin, and clindamycin, with rates of 89.65%, 86.2%, 82.75%, 37.93%, 34.48%, and 31.03%, respectively. Additionally, CDC^36^ recommended that *P. aeruginosa* strains emerge with phenotypic resistance patterns to various antimicrobial classes, mainly β-lactams, tetracycline, aminoglycosides, macrolides, and quinolones, causing complicated diseases and high mortality, with severe economic losses, due to high-cost treatment and control measures. Together, the antimicrobial susceptibility results recorded that *P*. *aeruginosa *isolates displayed higher antibiotic resistance from food and human origin. Moreover, similar results were obtained by Badr et al.^37^ revealed *P. aeruginosa* resistance to tetracycline, doxycycline, nalidixic acid, lincomycin, neomycin, trimethoprim-sulfamethoxazole, and chloramphenicol, and sensitivity to levofloxacin, norfloxacin, ciprofloxacin, colistin sulphate and gentamicin, and streptomycin. Furthermore, *P. aeruginosa*strains exhibited MDR between 1999 and 2009, as reported by Williams et al.^38^, who also found that approximately 86% of sepsis-related deaths in pediatric units were attributed to *P. aeruginosa*.

Our findings described that almost all *P. aeruginosa* isolates displayed phenotypic resistance against various antibiotics from eight antimicrobial groups. This was in contact with Woolhouse and Ward^39^, who recorded that *P. aeruginosa*isolates were likely resistant to various drugs as a result of the unselective practice of several antibiotics in food-producing animals and humans. Besides, Sebola et al.^40^ observed *P. aeruginosa* resistance to β-lactam classes, predominantly to penicillin and cephalosporins.

Another critical aspect of *P. aeruginosa* is the presence of numerous virulence and antimicrobial resistance genes within its genome, as well as its ability to form biofilms, which enhance its resistance and pathogenicity^41^. In our study, the PCR successfully amplified 396, 118, and 504 nucleotide fragments of the *tox*A, *exo*S, and *opr*L virulence genes of *P. aeruginosa*strains. Additionally, Rezaloo et al.^34^ showed that *exo*S, *las*A, *exo*U, *las*B, *plc*H, and *alg*D were the most virulence-detected genes from meat and meat products origin, with rates of 75.86%, 68.96%, 58.62%, 51.72%, 48.27%, and 44.82%, respectively, and this highlights that the consumption of such contaminated food products poses a serious public health threat. Furthermore, Badr et al.^37^ applied PCR for efficient recognition of the *opr*L gene at 504 bp fragments. Moreover, Al-Wrafy et al.^42^ described exotoxin A, designated as the *tox* A gene, which is a defensive component that plays a prospective role in *P. aeruginosa* pathogenicity through its cytotoxic activity. The virulence activity is essential to collaborate during *P. aeruginosa* establishment and biofilm activity, permitting further infection, and enhancing more pathogenic effects.

In addition, our results revealed successful amplification of 639, 786, 516, and 570 nucleotide fragments of the *erm*B, *pel*A, *bla*TEM, and *tet*A genes of *P. aeruginosa* strains. Similar findings found that *bla*DHA, *bla*CTX-M, and *bla*SHV were the main commonly identified resistance elements with rates of 93.1%, 83.65%, and 48.27%, respectively^34^. Similarly, Ibrahim et al.^33^ reported that *P. aeruginosa* harbored the resistance genes *bla*TEM, *bla*SHV, and *erm*B, with a multiple antibiotic resistance (MAR) index of 0.50.

It’s worth mentioning that the ERIC-PCR method is a rapid screening, PCR-based tool based on the number of ERIC sequences, as well as a genetic pointer for bacterial genotyping^43^. Therefore, ERIC PCR is a trustworthy, low-cost method for genotyping of *P. aeruginosa*^44^. Furthermore, Eladawy et al.^45^ applied ERIC-PCR for *P. aeruginosa* genotyping and revealed 99 different ERIC patterns with 70% similarity that were categorized into 8 main clusters, and thus indicated the significance of recognizing and regulating the possible causes of *P. aeruginosa* infections. Furthermore, an Iranian study tested 99 MDR *P. aeruginosa* strains by REIC-PCR, eight clusters, and 50 single genetic copies were produced, with high heterogeneity among isolates from burn infections^46^. Nevertheless, next-generation sequencing will permit capturing of the whole genome of the bacterium and offer a definitive purpose. However, this might not be available/possible under many conditions.

## Conclusion, limitations, and recommendations

*P. aeruginosa* is a noteworthy healthcare-associated pathogen in humans and food consumers, causing mortalities. This current study spotlights the relatively high recovery rate of *P. aeruginosa* in human, water, and many food samples, and thus suggests its threat in the aspect of public health. In addition, almost all *P. aeruginosa* isolates displayed high levels of drug resistance for many antimicrobial drugs that constitute a significant challenge to food safety and human consumers, as well as limiting the selection of effective antibiotics. The existence of diverse virulence genetic elements among screened *P. aeruginosa* isolates suggests their critical role in the pathogenicity and disease severity. Furthermore, our study showed the high polymorphism and genetic variety among *P. aeruginosa* isolates from food and human origin, with relatively different progenitors, which revealed different infection sources. Further surveys using phenotypic and genotypic approaches are mandatory to explore the relationship between *P. aeruginosa* phenotypic resistance, virulence, and biofilm activity. Also, a better understanding of the epidemiology of antibiotic resistance transmission to humans would help to reduce the transmission of *P. aeruginosa* infections between patients and the environment and food-borne outbreaks. Conversely, whole-genome sequencing of *P. aeruginosa* is essential for accurate typing of clinical isolates; however, it remains unavailable in many laboratories.

Finally, this study highlights the importance of continuous surveillance to detect biofilm-forming and antimicrobial-resistant *P. aeruginosa* isolates from food sources. We recommend enhancing hygiene and sanitation practices throughout the food production and handling chain, as well as routine monitoring of high-risk foods and food-contact surfaces. Implementation of robust antimicrobial stewardship programs is also essential to reduce potential public health risks. Future research should involve larger sample sizes and broader molecular characterization to better understand the epidemiology, resistance mechanisms, and potential transmission pathways of food-associated *P. aeruginosa*. In hospital settings, strict infection control measures, including environmental sanitation, water system monitoring, adherence to hand hygiene protocols, antimicrobial stewardship, and routine surveillance, are critical to limit the spread of *P. aeruginosa* and reduce healthcare-associated infections. We also acknowledge that our hospital sampling, limited to surface swabs, may underestimate the true prevalence of *P. aeruginosa*.

## Materials and methods

### Ethics approval and consent to participate

Human samples and hospital surface swabs were collected by medical staff at Sadat City Hospital in Menoufia Governorate, which reviewed and approved the study, in accordance with the regulations of the Egyptian Ministry of Health and under full medical supervision. Written informed consent was obtained from all patients prior to specimen collection. In addition, various food samples, including water, fish, chicken, meat, and milk, were collected from local markets in the study area around the hospital. All animal-handling procedures, as well as samples’ collection and disposal, were performed according to the regulations of the Institutional Animal Care and Use Committee (IACUC) with oversight of the faculty of Veterinary Medicine, University of Sadat City, Egypt (Ethical approval number: VUSC-010-1-25).

### Study area

This studied area is Sadat City, which is one of the Menoufia Governorate centers, and considered as common of the industrialized areas in Egypt. The area is located about 94 km northwest of Cairo, between 30.3811◦ N and 30.5266◦ E, and it is considered one of the first-generation new urban publics in Egypt. Most inhabitants reside in rural areas^47^. The area is considered one of the manufacturing areas where the government emphasizes farming, factories of food manufacturing, and packaging. Therefore, the food industry is a major business for the residents in the study area.

### Samples collection, preparation, and processing

Fifty samples from each (water, minced meat, chicken, milk, fish, hospital surface, and human pus from open wounds), with a total of 350 samples, were gathered from December 2022 to April 2023. The selection of 50 samples per category was primarily guided by sample availability during the study period and practical feasibility, while ensuring equal representation across all sample types to allow meaningful comparative analysis.

Fish and chicken used in this study were purchased from local markets in Sadat City, Egypt. All food product samples were collected using a systematic random sampling approach from multiple local markets across the study area to enhance representativeness and minimize selection bias. The samples were placed in clean polyethylene plastic bags under a septic condition and transported in cooled conditions at 4 °C in a heat-insulated ice box as recommended by APHA^48^. While the human samples were collected from hospitals by medical staff under physician supervision, as recommended by Rice et al.^49^. Following, the samples were transported to the laboratory in cooled conditions at 4 °C for further microbiological laboratory investigation.

Each gram of samples/ml was transported into 10 ml of a sterile buffer peptone water supplemented with Novobiocin 10 mg/500 ml, then homogenized, and incubation was done at 37 °C for 18 ± 2 h under suitable conditions as described by APHA^48^. The human pus samples were further processed, homogenized, and incubated at 37 °C for 18 h in nutrient broth, as follows by Rice et al.^49^.

### Isolation and identification of *P. aeruginosa*

All the prepared specimens were streaked on selective medium Cetrimide-nalidixic acid agar within 8 h and incubated at 37 °C for 24 h. After 24 h incubation, the identified colonies were then subcultured on citramide agar for 24 ± 2 h at 37 ᵒC^50^. The positive colonies were then picked up for further subculturing on nutrient agar for the purification of colonies. The pure colonies were confirmed by morphological and biochemical identification, including oxidase test, Indole, Citrate, TSI, Catalase, H2S production, and Haemolytic activity according to Cheesbrough^51^.

### Antimicrobial profile of *P. aeruginosa*

The method of Kirby–Bauer disk diffusion was used for the recovered *P. aeruginosa* isolates to evaluate their antimicrobial susceptibility. Adjustment to 0.5 McFarland was used for the prepared bacterial suspension. All antimicrobial disks of different antimicrobial classes (Oxoid Ltd., UK) were used for this test.

The following antibiotics were used: 30 µg Oxytetracycline (TE), 30 µg Doxycycline (DO), 5 µg Ofloxacin(O), 5 µg Enrofloxacin (EN), 10 µg Norfloxacin (NOR), 5 µg Ciprofloxacin (CIP), 15 µg Erythromycin (E), 30 µg Amikacin (AK), 30 µg Kanamycin (K), 30 µg Chloramphenicol (C), 10 µg Apramycin (A), 10 µg Imipenem (IPM), 30 µg Amoxicillin (AX), 30 µg Cephradine (CE), 25 µg Sulfamethoxazole (SXT), and 25 µg Colistin (CO). The antimicrobial results were assessed according to the guidelines of CLSI^52^ by measuring the inhibition zone and recording it as resistant, intermediate, or susceptible.

### Molecular identification of *P. aeruginosa* isolates

The DNA of *P. aeruginosa* isolates was extracted using the commercial kits QIAamp DNA Mini Kit (Qiagen, Hilden, Germany). Using specific primers, some virulence and resistance genes of *P. aeruginosa* isolates were detected, as listed in Table 5. The PCR reaction was performed in a 50 µL volume composed of 25 µL of PCR Master Mix (Qiagen, Germany), 6 µL target DNA, 1 µL of each primer (10 pmol/µL), and deionized water was completed to up to 50 µL. The PCR reaction was conducted in a thermal cycler (Applied Biosystems, Foster, CA, USA). The following protocol was used: Amplification conditions composed of an initial denaturation at 94 °C for 5 min, followed by 35 cycles of amplification (secondary denaturation at 94 °C for 30 s, annealing at 50 °C for 40 s, and extension at 72 °C for 45 s) and final extension at 72 °C for 10 min. After that, 1.5% gel electrophoresis was prepared for the PCR products (15 µL) and examined under UV light.

### Genotyping and analysis of *P. aeruginosa* by enterobacterial repetitive intergenic consensus polymerase chain reaction (ERIC-PCR)

ERIC fingerprinting data were transformed into a binary code depending on the presence or absence of each band. For the construction of dendrograms, both the unweighted pair group method with arithmetic average (UPGMA) and Ward’s hierarchical clustering routine were applied. Also, cluster and dendrogram analyses were performed using SPSS, version 22^53,54^. Similarity index between examined specimens was calculated using the online search tool (https://planetcalc.com/1664/*).*

**Table 1:** Isolation rate of *P. aeruginosa* recovered from food and human samples.

Source	Total sample	Positive sample On Cetrimide agar	% of positive sample
Human	50	13/50	26%
Water	50	9/50	18%
Fish	50	9/50	18%
Chicken	50	6/50	12%
Meat	50	5/50	10%
Milk	50	8/50	16%
Surface	50	0/50	0%
Total	350	50/350	14.28%

**Table 2:** Antimicrobial profile of *P. aeruginosa* isolates from food products and human samples.

Antimicrobial agent	S		I		*R*	
	NO	%	NO	%	NO	%
Amoxicillin (AMX)	Zero	Zero	Zero	Zero	50	100
Erythromycin (E)	Zero	Zero	Zero	Zero	49	98
Cephradine (CE)	2	4	3	6	45	90
Colistin (CO)	5	10	3	6	42	82
Oxytetracycline (T)	11	22	2	4	37	74
Chloramphenicol (C)	14	28	1	2	35	70
Doxycycline (DO)	15	30	Zero	Zero	35	70
Kanamycin (K)	18	36	1	2	31	62
Ciprofloxacin (CP)	26	52	2	4	22	44
Ofloxacin (O)	30	60	1	2	19	38
Enrofloxacin (EN)	32	64	1	2	17	34
Sulphamethoxazol (SXT)	34	68	3	6	13	26
Norfloxacin (NOR)	39	78	2	4	9	18
Amikacin (AK)	45	90	1	2	4	8
Apramycin (A)	47	94	1	2	2	4
Imipenem (IPM)	48	96	1	2	1	2

% value was calculated according to the number of total tested isolates (*n* = 50).

**Table 3:** Profile of phenotypic drug resistance of *P. aeruginosa* from human and food samples.

Number of isolates	Number of classes	Antimicrobial resistance profile	MAR index
1 (2%)	10	CE, IPM, AMX, EN, O, CP, A, NOR, AK, SXT, DO, E, T, C, CO, K	1
1 (2%)	9	T, DO, AMX, CP, EN, NOR, CO, C, CE, K, A, AK, SXT, E, O	0.937
11 (22%)	8	T, C, DO, NOR, AK, AMX, SXT, K, CO, O, E, EN, CE, CP	0.875
9 (18%)	7	DO, T, CP, O, AMX, CO, C, K, EN, E, CE	0.688
9 (18%)	6	DO, C, CO, AMX, K, T, E, CE	0.500
4 (8%)	5	DO, T, CO, C, CE, AMX, E	0.437
2 (4%)	4	T, AMX, CO, CE, E	0.313
5 (10%)	3	CE, CO, AMX, E	0.250

MAR index was estimated as follows = No. of resistance/Total No. of tested antibiotics*.

MAR index = No. of resistance (Isolates classified as intermediate were considered sensitive for the MAR index)/Total No. of tested antibiotics.

**Table 4:** Prevalence of virulence and resistance genes in the tested isolates from different sources.

Serial number	Source	*erm*B	*pel*A	*tox*A	*opr*L	*exo*S	*bla*TEM	*tet*A
1	Human pus	**+**	**+**	**+**	**+**	**+**	**+**	**+**
2	Human pus	**-**	**+**	**+**	**+**	**+**	**+**	**+**
3	Human pus	**+**	**+**	**+**	**+**	**+**	**+**	**+**
4	Human pus	**-**	**+**	**+**	**+**	**+**	**+**	**+**
5	Human pus	**+**	**+**	**+**	**+**	**+**	**+**	**+**
6	Tap Water	**-**	**+**	**+**	**+**	**+**	**+**	**+**
7	Tap Water	**+**	**+**	**+**	**+**	**-**	**+**	**+**
8	Fish swab	**+**	**+**	**+**	**+**	**+**	**+**	**+**
9	Fish swab	**-**	**-**	**+**	**+**	**+**	**+**	**+**
10	Fish swab	**+**	**+**	**+**	**+**	**-**	**+**	**+**
11	Chicken meat	**+**	**+**	**+**	**+**	**+**	**+**	**+**
12	Minced Meat	**+**	**+**	**+**	**+**	**-**	**+**	**+**
13	Raw milk	**+**	**+**	**+**	**+**	**+**	**+**	**-**
14	Raw milk	**-**	**+**	**+**	**+**	**+**	**+**	**+**
15	Raw milk	**+**	**+**	**+**	**+**	**-**	**+**	**-**

**Table 5:** Oligonucleotide primers were used for amplification of *tox*A, *opr*L, *exo*S, *erm*B, *pel*A, *bla*TEM, *tet*A, and ERIC-PCR.

Target	Sequence	Amplified product (bp)	Annealing Temperature	Reference
*tox*A	GACAACGCCCTCAGCATCACCAGC CGCTGGCCCATTCGCTCCAGCGCT	396	55˚C	^55^
*opr*L	ATG GAA ATG CTG AAA TTC GGC CTT CTT CAG CTC GAC GCG ACG	504	55˚C	^56^
*exo*S	GCGAGGTCAGCAGAGTATCG TTCGGCGTCACTGTGGATGC	118	55˚C	^57^
*erm*B	AAAAAGTACTCAACCAAATA ATTTAAGTACCGTTACT	639	55˚C	^58^
*pel*A	CATACCTTCAGCCATCCGTTCTT GCATTCGCCGCACTCAG	786	60˚C	^59^
*bla*TEM	TCAGCAATAAACCAGC AAAAGTACTCAACCAAATA	516	55˚C	^60^
*tet*A	ATTTAAGTACCGTTACT CAGCCATCCGTTCTTC	570	50˚C	^61^
ERIC- PCR	CGCATTCGCCGCACTCAG CGGT CAGTCCGTTTGTTC	Variable	50˚C	^19^

## Supplementary Information

Below is the link to the electronic supplementary material.

Supplementary Material 1

## Data Availability

Datasets generated during and/or analyzed during the current study are available from the corresponding author upon reasonable request.
