# Supplementary material for: Prevalence and genotyping of Pseudomonas aeruginosa from food and human sources
Source: Sci Rep. 2026 Feb 17;16:7179. doi: 10.1038/s41598-026-37559-y (PMC12920895; doi:10.1038/s41598-026-37559-y)
Supplement: Supplementary file 1 — Supplementary Material 1 [file 41598_2026_37559_MOESM1_ESM.docx]

**Supplementary Figures**


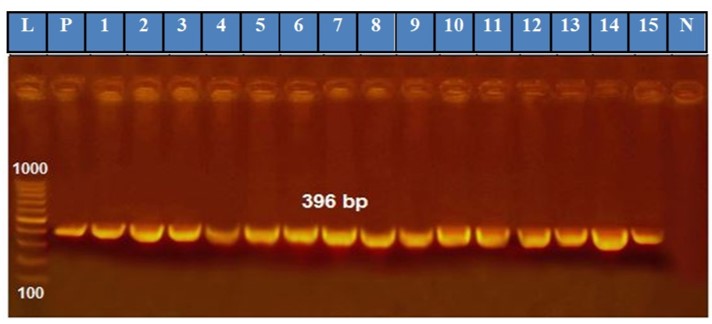


**Supplementary Fig. 1:** Agarose gel electrophoresis for PCR products that targeted the *Tox*A gene in *Pseudomonas aeruginosa* isolates using specific primers, and showing positive bands at 396 bp. Lane L: DNA ladder, lane P: Positive control, lane N: Negative control, and lanes 1-15: showing examined samples. (1-5): human, (6 and 7): water, (8, 9, and 10): fish, (11): chicken, (12): meat, and (13, 14, and 15): milk samples.


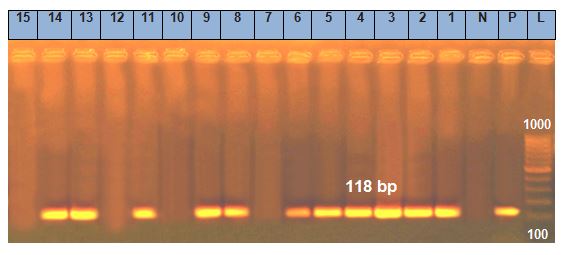


**Supplementary Fig. 2:** Agarose gel electrophoresis for PCR products that targeted the *exo*S gene in *Pseudomonas aeruginosa* isolates using specific primers, and showing positive bands at 118 bp. Lane L: DNA ladder, lane P: Positive control, lane N: Negative control, and lanes 1-15: showing examined samples described in Figure S1.


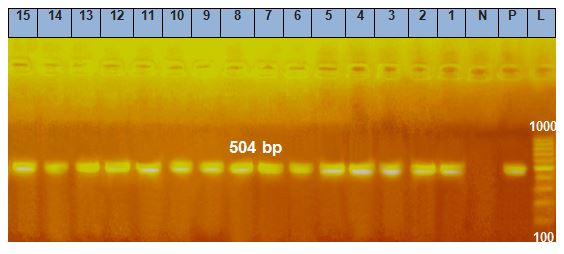


**Supplementary Fig. 3:** Agarose gel electrophoresis for PCR products that targeted the *opr*L gene in *Pseudomonas aeruginosa* isolates using specific primers, and showing positive bands at 504 bp. Lane L: DNA ladder, lane P: Positive control, lane N: Negative control, and lanes 1-15: showing examined samples described in Figure S1.


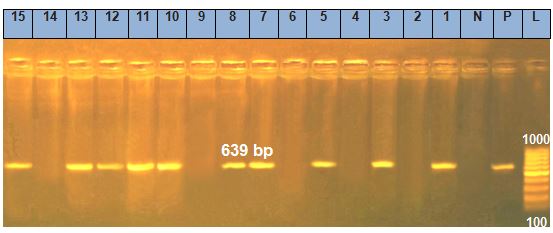


**Supplementary Fig. 4:** Agarose gel electrophoresis for PCR products that targeted the *erm*B gene in *Pseudomonas aeruginosa* isolates using specific primers, and showing positive bands at 639 bp. Lane L: DNA ladder, lane P: Positive control, lane N: Negative control, and lanes 1-15: showing examined samples described in Figure S1.


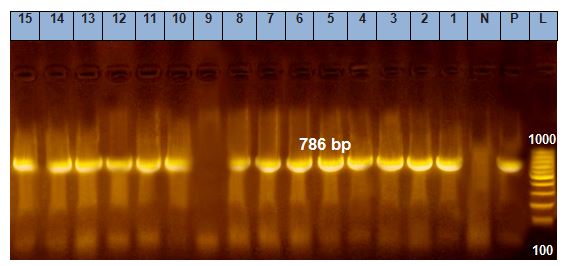


**Supplementary Fig. 5:** Agarose gel electrophoresis for PCR products that targeted the *pel*A gene in *Pseudomonas aeruginosa* isolates using specific primers, and showing positive bands at 786 bp. Lane L: DNA ladder, lane P: Positive control, lane N: Negative control, and lanes 1-15: showing examined samples described in Figure S1.


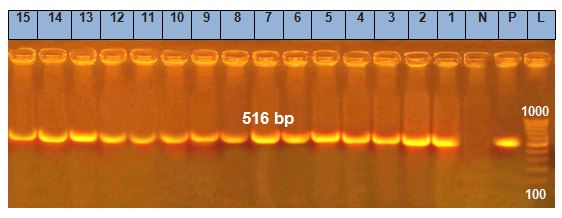


**Supplementary Fig. 6:** Agarose gel electrophoresis for PCR products that targeted the *bla*TEM gene in *Pseudomonas aeruginosa* isolates using specific primers, and showing positive bands at 516 bp. Lane L: DNA ladder, lane P: Positive control, lane N: Negative control, and lanes 1-15: showing examined samples described in Figure S1.


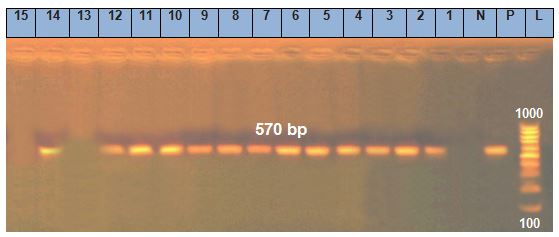


**Supplementary Fig. 7:** Agarose gel electrophoresis for PCR products that targeted the *tet*A gene in *Pseudomonas aeruginosa* isolates using specific primers, and showing positive bands at 570 bp. Lane L: DNA ladder, lane P: Positive control, lane N: Negative control, and lanes 1-15: showing examined samples described in Figure S1.


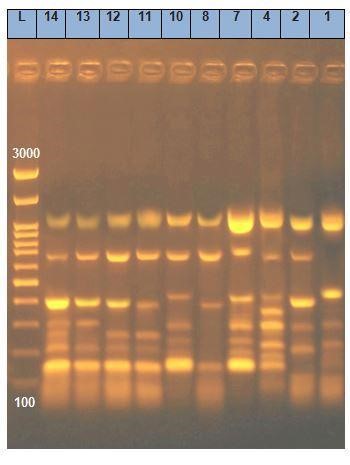


**Supplementary Fig. 8.** 1.5% Agarose gel electrophoresis of ERIC-PCR; Lane L: MDNA ladder, other lanes showing positive bands of the examined samples.
